# Interferon beta-1a for the maintenance of remission in patients with Crohn's disease: results of a phase II dose-finding study

**DOI:** 10.1186/1471-230X-9-22

**Published:** 2009-03-20

**Authors:** Claudia Pena Rossi, Stephen B Hanauer, Ratko Tomasevic, John O Hunter, Ira Shafran, Hans Graffner

**Affiliations:** 1 Merck Serono S.A. – Geneva, 15bis, Chemin des Mines, CH-1211 Geneva 20, Switzerland; 2 University of Chicago Medical Center, Chicago, Illinois, USA; 3 Clinical Hospital Center Zemun, Belgrade, Serbia; 4 Addenbrooke's NHS Trust, Cambridge, UK; 5 Shafran Gastroenterology Center, Winter Park, Florida, USA

## Abstract

**Background:**

Crohn's disease (CD) and multiple sclerosis (MS) share common pathogenic processes. Interferon (IFN) beta-1a is effective and generally well tolerated in patients with MS and has been shown to down-regulate the expression of interleukin-12, a cytokine that is thought to be involved in mucosal degeneration in CD. IFN beta-1a therefore offers promise as a treatment for CD.

**Methods:**

In this multicentre, double-blind, placebo-controlled, phase II, dose-finding study, patients with steroid-induced clinical remissions of CD were randomized 1:1:1:1 to subcutaneous IFN beta-1a: 66 mcg three times weekly (tiw), 44 mcg tiw, 44 mcg twice weekly (biw), or matching placebo tiw with steroid tapering. The primary endpoint was the proportion of patients relapse-free at Week 26. Safety was also assessed.

**Results:**

This study was terminated early following a planned interim analysis at 26 weeks. Of the planned 192 patients, 67 were randomized to treatment: placebo (n = 16), or IFN beta-1a 44 mcg biw (n = 17), 44 mcg tiw (n = 16) or 66 mcg tiw (n = 18). In total, 20/67 patients (29.9%) completed 26 weeks and 7 patients (10.4%) completed 52 weeks. The proportion of patients who remained relapse-free at Week 26 did not differ significantly between the placebo group (5/16, 31%) and the IFN beta-1a 44 mcg biw (6/17, 35%; p = 0.497), 44 mcg tiw (7/16, 44%; p = 0.280) or 66 mcg tiw (2/18, 11%; p = 0.333) groups. There was little difference between treatment groups in secondary efficacy endpoints. IFN beta-1a was generally well tolerated at all doses. Adverse events (AEs) were generally mild or moderate in IFN beta-1a-treated patients, with the most common AEs (influenza-like symptoms, headache, injection-site reactions) being similar to those reported with IFN beta-1a in MS.

**Conclusion:**

There was no difference in efficacy between patients with CD receiving IFN beta-1a or placebo. However, these results should be considered in the context of the low patient numbers and high dropout rate. Overall, IFN beta-1a was generally well tolerated, with a safety profile that was consistent with previous experience in MS.

**Trial registration:**

ClinicalTrials.gov NCT00304252

## Background

Crohn's disease (CD) is an inflammatory, autoimmune disease of the bowel that causes non-specific symptoms including abdominal pain, diarrhoea, anorexia and fatigue. The aetiology of CD is unknown [1] with environmental, immune, psychological and genetic factors believed to be involved [2,3]. CD is thought to arise primarily due to inappropriate chronic T-lymphocyte activation, with tissue damage induced by secondary macrophage activation [4,5]. The prospect that CD is a form of T-helper type 1 (Th1) cell-dominant autoimmune disease is gaining acceptance, with support from the current use of immunosuppressants [4,5].

There is currently no cure for CD, although various agents are used to induce and maintain remission and improve quality of life [6-8]. These include 5-aminosalicylic acid (5-ASA); antibiotics, such as ciprofloxacin and metronidazole; systemic or topical corticosteroids; and immunosuppressants, such as methotrexate and thiopurines (azathioprine, 6-mercaptopurine). There are, however, limitations associated with each of these conventional therapies, such as poor or unproven efficacy (5-ASA, antibiotics, azathioprine), potentially serious side-effects (corticosteroids, methotrexate), or a slow onset of action (thiopurines) [9]. More recently, infliximab and adalimumab (anti-tumour necrosis factor alpha) have been approved for use in patients with moderate-to-severe CD, natalizumab (anti-alpha-4 integrin) has been rejected in Europe for the treatment of CD (regulatory evaluation is ongoing in the USA) and a number of other biological therapies are in development for CD [10].

CD is an autoimmune condition characterized by a marked infiltration and accumulation of activated Th1-type CD4+ T cells and macrophages in inflamed intestinal mucosa, suggesting that the Th1 cell plays an important role in the pathogenesis of tissue damage in CD [4,5]. Studies indicate that the cytokine interleukin (IL)-12, the major Th1-inducing factor, contributes to the local inflammatory response in CD [11]. Indeed, studies consistently demonstrate that IL-12 levels are elevated in CD mucosa, and that boosting lamina propria Th1-cell responses with IL-12 produces severe mucosal degeneration [12-15]. Murine models have also shown that neutralization of IL-12 leads to a rapid and complete recovery of experimental colitis resembling CD [16]. Studies of anti-IL-12 antibodies in CD have reported beneficial effects [10].

Interferon (IFN) beta-1a, 44 mcg administered subcutaneously (sc) three times weekly (tiw) (Rebif^®^, Merck Serono S.A. – Geneva, Switzerland), is an approved treatment for patients with relapsing forms of multiple sclerosis (MS). Among other pathogenic targets, IFN beta has been shown to down-modulate T-cell activation by altering the expression of proteins involved in antigen presentation, and studies indicate that it also promotes the differentiation of activated T cells away from a Th1 response (pro-inflammatory) and towards a T-helper-type 2 (Th2) response (anti-inflammatory) [17]. Accordingly, IFN beta down-regulates the expression of IL-12 and promotes the expression of anti-inflammatory cytokines [17].

Given these mechanisms of action, and the consistent efficacy and tolerability of IFN beta-1a in patients with MS [18], it was of interest to discover whether IFN beta-1a could offer effective treatment for CD, with fewer side-effects and toxicity issues than existing treatments. A dose-finding study was conducted to investigate the efficacy and tolerability of IFN beta-1a in patients with CD. The standard dose used for the treatment of MS (44 mcg tiw) was compared with two doses lying on either side of the recommended weekly dosage regimen: one the same dose but less frequent (44 mcg twice weekly [biw]), and the other a higher dose with the same frequency (66 mcg tiw).

## Methods

This was a 52-week, multicentre (40 investigators in seven countries from North America and Europe), double-blind, placebo-controlled, phase II, dose-finding study (protocol 22916) of IFN beta-1a sc for the maintenance of remission in adult patients with CD. Written informed consent was obtained from each study participant before the initiation of the study.

### Ethical approval

The study was approved by the local ethics committees (ECs) or institutional review boards (IRBs), and conducted between December 2001 and September 2003 according to the principles of good clinical practice and with the ethical principles of the Declaration of Helsinki (2000) [19,20]. A complete listing of the ethics committees that granted approval for the study is listed below:

#### Belgium

Commissie Voor Medische Ethiek/Klinisch Onderzoek, Leuven

#### Germany

Ethikkommission der Landesärztekammer Westfalen-Lippe, Münster; Ethikkommission der Medizinischen Fakultat der CAU, Kiel; Ethikkommission der Landesärztekammer Schleswig-Holstein, Bad Segeberg

#### Italy

Comitato Etico Indipendente Locale, Azienda Ospedaliera "Ospedale Policlinico Consorziale" di Bari, Bari

#### Norway

Regional Etikk Komite Sor, Oslo

#### Serbia

Ethics Committee and Committee for Drugs, Military Medical Academy, Belgrade; Clinical Centre Serbia Ethical Committee, Belgrade; Klinicki Centar Zemun Eticki Komitet, Beograd-Zemun

#### Sweden

Research Ethical Committee, Department of Medicine-Odontology, Umeå University, Umeå

#### United Kingdom

Huntingdon Local Research Ethics Committee, Papworth Hospital, Cambridge; Cambridge Local Research Ethics Committee, Addenbrooke's NHS Trust, Cambridge; Rotherham Health Authority Local Research Ethics Committee, Rotherham.

### Study objectives

The primary objective was to determine the optimal dose of IFN beta-1a, based on safety and efficacy, for maintaining remission of CD in patients in whom remission had been achieved using steroids in the 4 weeks prior to study entry. Secondary objectives included determining the effect of IFN beta-1a on the time to relapse, patients' quality of life (using the Inflammatory Bowel Disease Questionnaire [IBDQ]), Crohn's Disease Activity Index (CDAI) score, biological markers of inflammation, fistulas and antibodies to IFN beta-1a.

### Patients

IFN beta-naïve patients aged 18 years or older were included if they had an established diagnosis of CD by standard criteria (i.e. endoscopy, histology, barium contrast radiograph of gastrointestinal tract, or other recognized criteria) and had gone into remission, defined as a decrease in the CDAI to ≤150 points, using corticosteroids within 4 weeks prior to study day 1 (SD1). On SD1, patients were to be receiving ≥20 mg/day and ≤40 mg/day of prednisone or its equivalent, or 6–9 mg/day of budesonide, and to be able to comply with and tolerate the steroid-tapering schedule. Steroid tapering was performed over a maximum of 11 weeks (depending on dose at study entry), as follows. Prednisone (or prednisone equivalent) dosage was reduced on a weekly basis in increments of 5 mg/day down to a dosage of 10 mg/day, and thereafter by weekly increments of 2.5 mg/day, until complete withdrawal. Budesonide dosage was reduced by 3.0 mg/day at 3-weekly intervals from 9.0 mg/day to 3.0 mg/day, followed by complete withdrawal after 4 weeks of the 3.0 mg/day dose. Patients were not to have been taking anti-inflammatory agents, immunosuppressants, immunomodulators and anti-cytokines for >2 weeks preceding the day of study screening, and they were not to have been receiving any other therapy for the maintenance of remission in CD. Patients were required to have adequate bone marrow reserve (defined by white blood cell count ≥3.5 × 10^9^/L, neutrophils ≥1.5 × 10^9^/L, thrombocytes ≥100 × 10^9^/L and haemoglobin ≥8.5 g/dL), adequate liver function (defined by total bilirubin, aspartate aminotransferase, alanine aminotransferase or alkaline phosphatase levels <2 times the upper limit of normal) and adequate renal function (defined by serum creatinine level <2.0 mg/dL). Female patients had to be post-menopausal, surgically sterilized or to have agreed to use appropriate contraception for the duration of the study, and to have been neither pregnant nor breast-feeding.

Patients were excluded if they required maintenance treatment for the management of CD or had participated in any other investigational study or received an experimental therapeutic procedure considered to interfere with the study within 3 months of SD1. Patients could not have had a stoma, symptoms of intestinal stenosis regarded mainly as non-inflammatory, active infection, or a history of cancer or systemic disease that could have interfered with patient safety, compliance or evaluation of CD. Patients were also excluded if they needed emergency surgery (for uncontrollable haemorrhage, persistent non-inflammatory intestinal obstruction or perforation), had scheduled elective surgery (during the study period) or had surgery in the 4 weeks preceding SD1, or if they had an unstable psychiatric disorder, severe depressive disorder and/or suicidal ideation, epilepsy with a history of seizures not adequately controlled by treatment, or allergies to paracetamol, human serum albumin or mannitol.

Two main populations were analysed in this study: a modified intention-to-treat (ITT) population for efficacy parameters and an 'all treated patients' population for safety parameters. The modified ITT population comprised all randomized patients who received ≥1 injection of the study medication and had data from ≥1 post-SD1 visit. A modified ITT population was used because 1 patient in the placebo group had received IFN beta-1a 66 mcg tiw in error throughout the study. Data for this patient are presented according to the treatment received and not the randomized treatment group. The 'all treated patients' population included patients who were randomized and who received ≥1 injection of study medication.

### Treatment

Patients underwent randomization (1:1:1:1) to one of four groups: IFN beta-1a 66 mcg tiw, IFN beta-1a 44 mcg tiw, IFN beta-1a 44 mcg biw or matching placebo tiw (same excipients but no IFN beta-1a). Treatment was to be self-administered by sc injection for a total of 52 weeks. Randomization took place within 24 hours prior to the first dose of study medication, according to a computer-generated randomization list stratified by geographical region, which was allocated through a centralized telephone randomization system.

The efficacy and safety assessments carried out at each visit from SD1 to 4 weeks following the last injection are summarized in Table 1.

**Table 1:** Summary of efficacy and safety assessments carried out throughout the study

	Treatment period (weeks)											Post-treatment period (weeks)
	**SD1**	**2**	**6**	**10**	**14**	**18**	**22**	**26**	**34**	**42**	**52**	**4**

Efficacy and safety assessments*		X	X	X	X	X	X	X	X	X	X	X
IBDQ assessment	X							X			X	
CDAI assessment	X	X	X	X	X	X	X	X	X	X	X	
CRP	X		X		X		X	X	X	X	X	X
ESR	X		X		X		X	X	X	X	X	X
Antibodies to IFN beta-1a	X							X			X	
Thyroid function	X							X			X	
Urinalysis	X							X			X	X
Pregnancy testing	X			X		X		X			X	

CDAI: Crohn's disease activity index; CRP: C-reactive protein; ESR: erythrocyte sedimentation rate; IBDQ: inflammatory bowel disease questionnaire; IFN, interferon; SD1, study day 1

*Safety data were obtained by monitoring adverse events/serious adverse events, concomitant medications, physical examinations, vital signs and routine analysis of blood (haematology and chemistry)

### Efficacy measures

The primary efficacy endpoint was the proportion of patients who maintained remission (relapse-free) and did not receive any additional treatment for the management of CD by Week 26. Relapse was defined by an increase in the CDAI to a total score of ≥200 points at any time, and by an increase in the CDAI of >70 points from SD1 measured at two consecutive visits.

Secondary efficacy endpoints assessed the effect of treatment with IFN beta-1a on the following: the proportion of patients who maintained remission (relapse-free) and did not receive any additional treatment for the management of CD by Week 52; time to relapse; and change from SD1 to end of treatment in the quality-of-life (IBDQ) score, CDAI score, biological markers of inflammation (C-reactive protein and erythrocyte sedimentation rate), number of fistulas (including new fistulas and closure of existing ones) and antibodies to IFN beta-1a. Blood samples taken at SD1, Week 26 and Week 52 were tested for the presence of binding antibodies (BAbs) and, if found positive (defined as a titre ≥20 neutralizing units [NU]/mL), were assessed for neutralizing activity. Patients with a neutralizing antibody (NAb) titre of ≥20 NU/mL were considered to have a positive result for NAbs.

### Safety measures

Safety endpoints included the incidence of adverse events (AEs) and serious AEs, and changes in clinical and laboratory parameters from SD1.

**Figure 1:**
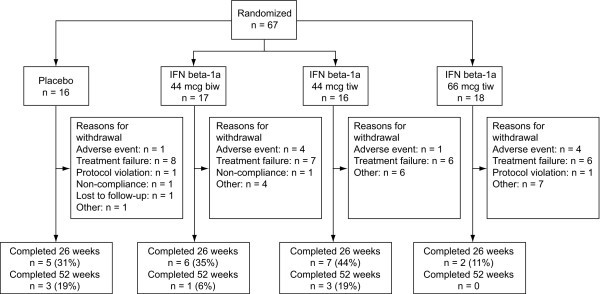
**Patient disposition**. biw: twice weekly. IFN: interferon. tiw: three times weekly.

Interim analysis of the secondary endpoints was added as an amendment to the protocol in January 2003 because the study had been recruiting slowly and patient dropout rates had been high (Figure 1). This analysis was to allow for early discontinuation of the study in the case of lack of efficacy.

### Statistical analysis

All statistical analyses were performed using SAS^® ^Version 6.12 software (SAS Institute Inc.; Cary, NC, USA). A sample size of 43 patients per treatment group was needed to provide 70% power to detect a significant difference in the primary endpoint. This calculation was performed using a two-sided chi-squared test and assumed a type I error rate of 10%. Calculations were based on the assumption that the proportion of patients who maintained remission and did not receive additional treatment for CD at 26 weeks in the placebo group would be 25% and would be at least 50% in an effective interferon beta-1a group.

All data were summarized using descriptive statistics. The difference between groups was assessed using analysis of variance on raw or ranked data (demographics and CDAI at SD1), a logistic regression model (primary and secondary efficacy endpoints) or a Cox model (time to relapse) with effects for treatment and region. All tests were performed at a 10% significance level. Because of the small number of patients in each treatment group, the differences between groups were not analysed for the change from baseline (SD1) in a number of the secondary endpoints (IBDQ score, CDAI score, biological markers of inflammation, antibodies to IFN beta-1a and number of fistulas). Continuous (quantitative) variables were tabulated using the following summary statistics: number of values, mean, standard deviation (SD), and median and range (minimum to maximum). Discrete (qualitative/categorical) variables were tabulated using frequencies and percentages. An interim analysis of study data was performed at Week 26 and used to assess study futility. This used the method of O'Brien-Fleming to adjust for the overall type I error level when analysing secondary efficacy variables.

## Results

### Patients

This study was terminated early due to lack of efficacy, following an interim analysis of the 26-week data. In all, 67 of the planned 192 patients underwent randomization. Of these, 16 received placebo, 17 received IFN beta-1a 44 mcg biw, 16 received IFN beta-1a 44 mcg tiw and 18 received IFN beta-1a 66 mcg tiw (Figure 1). Table 2 summarizes the key baseline (SD1) demographic information and clinical characteristics of the patients recruited. More than 93% of patients in each treatment group (97% overall) were white, with the remainder Hispanic. Patient demographic and clinical characteristics were well balanced, with no statistically significant differences between the four treatment groups. Overall, the diagnosis of CD was confirmed most frequently by endoscopy (56/67, 84%) and histology (44/67, 66%). In all, 51% of patients (34/67) had CD on the left side of the colon, 51% (34/67) had CD on the right side of the colon, and 69% (46/67 patients) had CD in the ileum. The majority of patients had not undergone surgery for their CD, although approximately half had undergone some other procedure (52%; 35/67 patients). The mean ± SD CDAI score at baseline was highest for the placebo group (100.8 ± 47.1) and lowest for the IFN beta-1a 66 mcg tiw group (79.2 ± 30.0). There was no significant difference in baseline CDAI score among the treatment groups. Additional baseline characteristics summarized included those assessed for secondary endpoints, past and current medical conditions, smoking history, electrocardiographic findings, individual variables of the CDAI and duration of CD. There were no significant differences among the treatment groups for any of these additional baseline characteristics.

**Table 2:** Baseline demographic data and clinical characteristics*

Characteristics	Placebo (n = 16)	IFN beta-1a 44 mcg biw (n = 17)	IFN beta-1a 44 mcg tiw (n = 16)	IFN beta-1a 66 mcg tiw (n = 18)	Total (n = 67)
Mean (SD) age (years)	37.6 (13.6)	42.1 (14.8)	39.4 (12.9)	35.1 (11.9)	38.5 (13.3)
Mean (SD) BMI (kg/m^2^)	23.5 (7.8)	24.2 (6.4)	24.4 (4.7)	24.3 (5.8)	24.1 (6.1)
Sex					
Male	8 (50)	6 (35.3)	7 (43.8)	7 (38.9)	28 (41.8)
Female	8 (50)	11 (64.7)	9 (56.3)	11 (61.1)	39 (58.2)
Race					
White	15 (93.8)	17 (100)	16 (100)	17 (94.4)	65 (97)
Hispanic	1 (6.3)			1 (5.6)	2 (3)
Diagnosis confirmed by:	14 (87.5)	15 (88.2)	12 (75)	15 (83.3)	56 (83.6)
Endoscopy	11 (68.8)	11 (64.7)	12 (75)	10 (55.6)	44 (65.7)
Histology	6 (37.5)	8 (47.1)	9 (56.3)	10 (55.6)	33 (49.3)
Barium contrast radiography	4 (25)	4 (23.5)	6 (37.5)	4 (22.2)	18 (26.9)
Other					
Surgery					
Yes	5 (31.3)	3 (17.6)	2 (12.5)	4 (22.2)	14 (20.9)
No	11 (68.8)	14 (82.4)	14 (87.5)	14 (77.8)	53 (79.1)
Mean CDAI total score	100.8	92.1	80.4	79.2	87.9

biw: twice weekly; BMI: body mass index; CDAI: Crohn's Disease Activity Index; IFN: interferon; SD, standard deviation; tiw: three times weekly

*Data are presented as number (%) unless indicated otherwise; percentages may not sum to 100 due to rounding

A total of 20 patients (29.9%) completed 26 weeks and 7 patients (10.4%) completed 52 weeks of the study. Of the 60 patients who discontinued prior to 52 weeks, the reasons for discontinuation were: treatment failure (40%, 27/67 patients); AEs (15%, 10/67 patients); major protocol violation (3.0%, 2/67 patients); non-compliance (3.0%, 2/67 patients); lost to follow up (1.5%, 1/67 patients); or 'other reasons' (27%, 18/67 patients). One patient who had undergone randomization to the placebo group received IFN beta-1a 66 mcg tiw in error throughout the study. This patient is presented in all tables and figures according to the treatment received and not the randomized treatment group. Nine patients had inclusion or exclusion criteria deviations at study entry; none of these were considered major protocol deviations. Five of these patients were granted exceptions and allowed to continue in the study. The remaining 4 patients (2 in the placebo group, 1 in the IFN beta-1a 44 mcg tiw group and 1 in the IFN beta-1a 66 mcg tiw group) had not gone into remission using corticosteroids within the 4 weeks prior to SD1. These deviations were not discovered until after the patients had started treatment and, although exceptions were not granted, these patients continued to participate in the study.

### Efficacy

**Figure 2:**
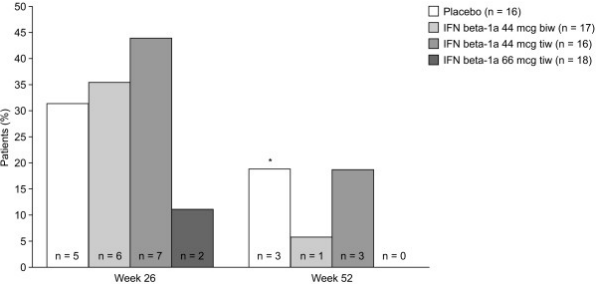
**Proportion of patients who maintained remission (relapse-free) at Week 26 or Week 52 and did not receive additional treatment for Crohn's disease**. biw: twice weekly; IFN: interferon; tiw: three times weekly. *p < 0.05 in favour of placebo over IFN beta-1a 66 mcg tiw at Week 52.

With respect to the primary outcome, there were no significant differences in the proportion of patients who remained relapse-free without additional treatment at Week 26 between the placebo group (5/16, 31%) and the IFN beta-1a 44 mcg biw (6/17, 35%; p = 0.497), 44 mcg tiw (7/16, 44%; p = 0.280) or 66 mcg tiw (2/18, 11%; p = 0.333; Figure 2) groups.

At Week 52, 0/18 patients in the IFN beta-1a 66 mcg tiw group remained relapse-free, compared with 3/16 patients (19%) in the placebo group (p = 0.043; Figure 2). The proportion of patients remaining relapse-free at Week 52 in the IFN beta-1a 44 mcg biw (1/17 patients, 6%; p = 0.330) and 44 mcg tiw (3/16 patients, 19%; p = 0.762) groups did not differ significantly from the placebo group. There were no significant differences between treatment groups in any of the other secondary endpoints.

Analysis using imputed data showed that there were no statistical differences between placebo and any of the IFN beta-1a treatment groups in time to relapse. All treatment groups showed a worsening of mean IBDQ and CDAI scores from SD1 to Week 52. The placebo group had a smaller mean increase in CDAI from SD1 to the end of Week 26 (mean [SD] increase: 0.8 [25.3]) than the IFN beta-1a treatment groups (mean [SD] increase for IFN beta 1a, 44 mcg tiw: 9.7 [49.5]; 44 mcg biw: 11.6 [70.3]; 66 mcg tiw: not reported).

The increase in mean ± SD erythrocyte sedimentation rate from SD1 to end of treatment was smaller for the placebo group (3.0 ± 12.2 mm/hr) than for any of the IFN beta-1a treatment groups (range 15.3 ± 40.0 to 22.7 ± 22.3 mm/hr). All treatment groups showed increases in C-reactive protein level by the end of treatment, with the greatest mean increase observed in the IFN beta-1a 44 mcg tiw group. For patients with fistulas at the end of the study (2 patients in the placebo group, 3 in the IFN beta-1a 44 mcg tiw group and 1 in the IFN beta-1a 66 mcg tiw group), there were no changes from SD1 to end of treatment in the number of fistulas.

At SD1, 1/17 patients (5.9%) in the IFN beta-1a 44 mcg biw group and no patients in all other treatment groups had a positive result for BAbs to IFN beta-1a. At Week 26, 4/16 patients (25.0%) in the IFN beta-1a 44 mcg tiw group had positive BAb results, and at the end of treatment positive BAbs results were found in 4/17 patients (23.5%) in the IFN beta-1a 44 mcg biw group, 7/16 patients (43.8%) in the IFN beta-1a 44 mcg tiw group and 5/18 (27.8%) patients in the IFN beta-1a 66 mcg/tiw group. No patients in the placebo group had a positive result at Week 26 or at the end of treatment. Samples testing positive for BAbs were also tested for NAbs. Of the patients who were BAb-positive, 3 also tested positive for NAbs; all 3 had received IFN beta-1a 44 mcg tiw.

### Safety outcomes

A total of 452 AEs were reported in 62 (93%) of the 67 patients enrolled. The most common AEs were influenza-like symptoms (24/67 patients; 36%), headache (22/67 patients; 33%) and injection-site reactions (18/67 patients; 27%) (Table 3). The majority of AEs were mild or moderate in severity.

**Table 3:** Summary of the most frequent adverse events that occurred during the study (frequency ≥ 10% of patients)

	Number of patients (%)				
Adverse event (preferred term)	Placebo (n = 16)	IFN beta-1a 44 mcg biw (n = 17)	IFN beta-1a 44 mcg tiw (n = 16)	IFN beta-1a 66 mcg tiw (n = 18)	Total (n = 67)
Influenza-like symptoms	3 (18.8)	8 (47.1)	6 (37.5)	7 (38.9)	24 (35.8)
Headache	3 (18.8)	8 (47.1)	7 (43.8)	4 (22.2)	22 (32.8)
Injection-site reaction	2 (12.5)	5 (29.4)	6 (37.5)	5 (27.8)	18 (26.9)
Fever	2 (12.5)	3 (17.6)	5 (31.3)	6 (33.3)	16 (23.9)
Abdominal pain	4 (25.0)	2 (11.8)	5 (31.3)	5 (27.8)	16 (23.9)
Injection-site inflammation	2 (12.5)	3 (17.6)	5 (31.3)	5 (27.8)	15 (22.4)
Fatigue	3 (18.8)	3 (17.6)	4 (25.0)	2 (11.1)	12 (17.9)
Nausea	3 (18.8)	3 (17.6)	2 (12.5)	3 (16.7)	11 (16.4)
ESR increased	0	2 (11.8)	5 (31.3)	3 (16.7)	10 (14.9)
Arthralgia	2 (12.5)	4 (23.5)	3 (18.8)	1 (5.6)	10 (14.9)
Injection-site rash	0	3 (17.6)	3 (18.8)	2 (11.1)	8 (11.9)
Rhinitis	2 (12.5)	2 (11.8)	3 (18.8)	1 (5.6)_	8 (11.9)
Myalgia	1 (6.3)	4 (23.5)	2 (12.5)	1 (5.6)	8 (11.9)
Injection-site pain	0	2 (11.8)	1 (6.3)	4 (22.2)	7 (10.4)
Anaemia	0	4 (23.5)	2 (12.5)	1 (5.6)	7 (10.4)

biw: twice weekly; ESR: erythrocyte sedimentation rate; IFN: interferon; tiw: three times weekly

Eight severe AEs occurred in 4/16 patients (25.0%) in the placebo group (headache, night sweats, nausea, tenesmus, tooth disorder, increased creatine phosphokinase level, involuntary muscle contractions, tremor). There were 4 severe AEs in 4/17 patients (23.5%) receiving IFN beta-1a 44 mcg biw (bronchitis, arthralgia, myalgia and pulmonary embolism), 4 severe AEs in 4/16 patients (25.0%) receiving IFN beta-1a 44 mcg tiw (abdominal pain, tenesmus, myalgia, tachycardia) and 4 severe AEs in 3/18 patients (16.7%) receiving IFN beta-1a 66 mcg tiw (3 cases of abdominal pain and 1 case of dehydration). There were no life-threatening AEs.

No deaths were reported during this study. There were 4 serious AEs in 4 patients (2 events in the placebo group, 1 in the IFN beta-1a 44 mcg biw group and 1 in the IFN beta-1a 66 mcg tiw group). Two of the events – severe pulmonary emboli in a patient treated with IFN beta-1a 44 mcg biw and moderate cellulitis of the ear (infection) in a patient treated with IFN beta-1a 66 mcg tiw – were considered 'possibly' related to study treatment. The other 2 events – moderate dyspnoea on exertion and moderate lower gastrointestinal bleed (both in patients in the placebo group) – were considered 'unlikely' to be related to study treatment. Ten patients withdrew from the study because of AEs: 1 in the placebo group, 4 in the IFN beta-1a 44 mcg biw group, 1 in the IFN beta-1a 44 mcg tiw group and 4 in the IFN beta-1a 66 mcg tiw group.

The incidence of AEs known or suspected to be associated with IFN therapy, such as influenza-like symptoms, injection-site reactions, hepatic abnormalities (particularly elevated transaminase levels), cytopenias and depression, was higher in the IFN beta-1a treatment groups than the placebo group, but was consistent with previous experience with IFN therapy. All these events were mild-to-moderate in severity.

In general, changes from SD1 in haematology, blood chemistry and urinalysis parameters were either expected effects of IFN beta-1a treatment or within the normal range, and thus were not considered clinically meaningful. No clear treatment- or dose-related effects on thyroid function were observed. There were no clinically significant changes from SD1 in vital signs over the course of the study. Steroid tapering was similar for the four treatment groups.

## Discussion

Following a 26-week interim analysis of data collected from 67 patients, this study demonstrated that IFN beta-1a does not have superior efficacy to placebo for maintaining remission in patients with CD. The study was, therefore, terminated prematurely. An amendment to the protocol permitted interim analysis of the secondary endpoints to allow for early study discontinuation in the case of lack of efficacy. There was little difference between treatment groups in terms of secondary endpoints. IFN beta-1a was generally well tolerated at all doses studied, including the highest dosage of 66 mcg tiw, and the overall safety profile was consistent with previous experience in patients with MS.

The most common AEs observed, such as influenza-like symptoms, headache and injection-site reactions, were more frequent in the IFN beta-1a treatment groups than in the placebo group. However, these AEs are among the most common side-effects seen with IFN beta-1a in patients with MS [21], and therefore effective strategies are already in place for their management. For example, influenza-like symptoms can be treated using non-steroidal anti-inflammatory drugs, such as ibuprofen, and novel injection devices and formulations have been developed to minimize injection-site reactions [22]. In this study, IFN beta-1a was administered at a higher dose than that used in MS. Importantly, the high dosage of 66 mcg tiw was also generally well tolerated and was associated with a similar incidence of AEs to the 44 mcg tiw dosage indicated in MS.

Despite the similarities between the pathogenic mechanisms of CD and MS, and the promising match between the mechanism of action of IFN beta-1a and potential therapeutic targets in CD, this study indicates that IFN beta-1a is ineffective for the treatment of CD at the doses evaluated. One explanation for the different effects in MS and CD may be the many pathogenic pathways that IFN beta is thought to influence in MS [17]. As well as down-modulating T-cell activation and differentiation to Th1-type CD4+ cells, IFN beta is also believed to inhibit activated Th1-cell trafficking across the blood-brain barrier and to prevent T-cell reactivation in the central nervous system [17]. If the efficacy of IFN beta in MS is due more to its effects on these last two processes (specific to MS) than on the induction of Th1-type CD4+ cells (important in MS and CD), this could explain its lack of efficacy in CD.

Although the percentage of patients relapse-free at Week 52 was significantly lower in the IFN beta-1a 66 mcg tiw group than in the placebo group, this finding was not seen with the other two IFN beta dosing regimens nor with the primary or other secondary endpoints. Furthermore, given the low patient numbers in this analysis, it is not possible to conclude that IFN beta-1a may worsen CD. Clinical experience with type I IFNs for the treatment of CD is very limited. Few studies have been reported, all of which included only a small number of patients and results were variable [23-25]. Similarly, clinical studies of IFN alpha or IFN beta in patients with ulcerative colitis (UC) have reported promising results [26-28] or a lack of efficacy [29,30]. Furthermore, isolated reports of UC in patients receiving IFN beta-1a for MS [31] or IFN alpha for hepatitis C infection [32,33] do not support a therapeutic role for type I IFNs in the treatment of inflammatory bowel disease.

Although the results reported here suggest that IFN beta-1a is not an effective treatment for CD, these results should be considered in the context of the low patient numbers and high drop-out rate. Only 67 patients of a planned recruitment target of 192 patients (35%) were randomized to treatment. Of these patients only 20 (10.4% of the target number) completed the study; notably, treatment failures were the most common cause of study drop-out. Thus, the findings reported here are based on a very small number of patients, as with other studies of IFNs in UC and CD.

Despite the lack of efficacy reported here, the results of this study do lend further support to the favourable safety profile of this treatment, which has already been demonstrated in patients with MS. Moreover, these results indicate that higher IFN beta-1a doses may offer a similar safety profile to the dose currently indicated in patients with MS.

## Conclusion

Overall, analyses showed no difference in efficacy between the IFN beta-1a treatment groups and placebo for maintaining remission in patients with CD. However, IFN beta-1a treatment was generally well tolerated, even at the highest dosage of 66 mcg tiw, further supporting the favourable safety profile of this treatment, both at the dose indicated for MS and at higher doses.

## Competing interests

CPR and HG are employees of Merck Serono S.A. – Geneva. At the time of the study, SBH was a consultant for Merck Serono S.A. – Geneva, who also provided his institution with clinical research funding for this trial. JOH, RT and IS have no competing interests.

## Authors' contributions

JOH, RT and IS were involved in the initial protocol design, patient recruitment and assessment. CPR and HG were involved in the evaluation of the data. All authors were involved in the writing of the manuscript. All authors have seen and approved the final manuscript.

## Pre-publication history

The pre-publication history for this paper can be accessed here:

http://www.biomedcentral.com/1471-230X/9/22/prepub
